# Circulating miRNA as potential biomarkers for diabetes mellitus type 2: should we focus on searching for sex differences?

**DOI:** 10.1007/s13353-021-00678-5

**Published:** 2022-01-05

**Authors:** Weronika Kraczkowska, Lucyna Stachowiak, Andrzej Pławski, Paweł Piotr Jagodziński

**Affiliations:** 1 Department of Biochemistry and Molecular Biology, Poznań University of Medical Science, 6 Święcickiego Street, 60-781 Poznan, Poland; 2 Institute of Human Genetics, Polish Academy of Sciences, 60-479 Poznan, Poland

**Keywords:** Diabetes mellitus type 2, miRNA, Circulating miRNA, Sex differences, Biomarkers

## Abstract

microRNAs are non-coding molecules, approximately 22 nucleotides in length, that regulate various cellular processes. A growing body of evidence has suggested that their dysregulated expression is involved in the pathogenesis of diverse diseases, including diabetes mellitus type 2 (DM2). Early onset of this chronic and complex metabolic disorder is frequently undiagnosed, leading to the development of severe diabetic complications. Notably, DM2 prevalence is rising globally and an increasing number of articles demonstrate that DM2 susceptibility, development, and progression differ between males and females. Therefore, this paper discusses the role of microRNAs as a source of novel diagnostic biomarkers for DM2 and aims to underline the importance of sex disparity in biomarkers research. Taking into account an urgent need for the development of sex-specific diagnostic strategies in DM2, recent results have shown that circulating miRNAs are promising candidates for sex-biased biomarkers.

## Introduction

International Diabetes Federation (IDF) projects that the worldwide prevalence of diabetes among adults (20–79 years old) will systematically rise to finally reach about 700 million people by 2045 (Saeedi et al. 2019). Notably, many cases of diabetes are undiagnosed until the appearance of complications (Association 2003), which may lead to severe disorders development such as cardiovascular diseases, renal failure, retinopathy, neuropathy, and even lower extremity amputations (Susan van et al. 2010; Schmidt 2018). This data shows that diabetes continues to be one of the most crucial public health issues (Saeedi et al. 2019). Diabetes mellitus type 2 (T2DM or DM2) represents the majority of all diabetes cases (Chatterjee et al. 2017). This chronic metabolic disorder is characterized by hyperglycemia along with insulin resistance and usually relative insulin deficiency (Association 2014). Currently, an increasing number of studies indicate that susceptibility and pathogenesis of DM2 are sex-biased (Kautzky-Willer et al. 2016). In general, the interplay of biological, environmental, social, or even lifestyle factors contributes to observed sex differences in DM2 (Kautzky-Willer et al. 2016). Moreover, the results of the clinical research from different populations confirm the significance of sex disparity in DM2. For example, comparative analysis in the European population showed that men develop DM2 at an earlier age and at a lower body mass index than women (Logue et al. 2011). A population-based study from Italy indicated a greater impact of diabetes on cardiovascular mortality in women (Ballotari et al. 2015). Recently, Li et al. 2021 demonstrated that the most common panel of anthropometric and biochemical parameters better predicts DM2 in women than in men from the Hainan population. In addition, utilizing conventional biomarkers such as glucose homeostasis-related parameters, lipid classes, and glycated hemoglobin displays some limitations such as not full specificity for DM2 and presence in subjects already suffering from metabolic disorders (Urdea et al. 2009; Bhatia et al. 2015). Given all the abovementioned reports, there is an urgent need to find novel diagnostic biomarkers, especially those indicating the early onset of DM2 and taking into account sex differences.

To date, many studies have proved that molecules classified as non-coding RNAs (ncRNAs) may serve as a valuable source of prognostic, diagnostic, and even predictive biomarkers for many human diseases (Borga et al. 2019). Non-coding RNAs play important role in a variety of fundamental biological processes such as development, metabolism, cell proliferation, and apoptosis; therefore, their aberrant expression is associated with the development of various diseases (Wang et al. 2019). Interestingly, the results of the Encyclopedia of DNA Elements project reported that many coding and non-coding transcripts are processed into steady-stable precursors of small non-coding RNA (Dunham et al. 2012). Notably, microRNAs (miRNA) are a group of small non-coding RNA, approximately 22 nucleotides in length (Bartel 2018), which exert an important regulatory role in cells usually by inhibiting the expression of genes at the post-transcriptional level (Bartel 2004). A growing body of literature shows that many miRNAs are dysregulated in patients with DM2 and may play role in pathological mechanisms underlying the development of diabetes complications (He et al. 2017; Shi et al. 2020). Therefore, in this article, we discuss the potential role of miRNA as a source of biomarkers for DM2. In addition, with the growing emphasis on sex as a relevant biological variable (Mannon et al. 2020), we also underscore the importance of sex differences in biomarkers research.

## miRNA biogenesis and association with DM2

**Fig. 1:**
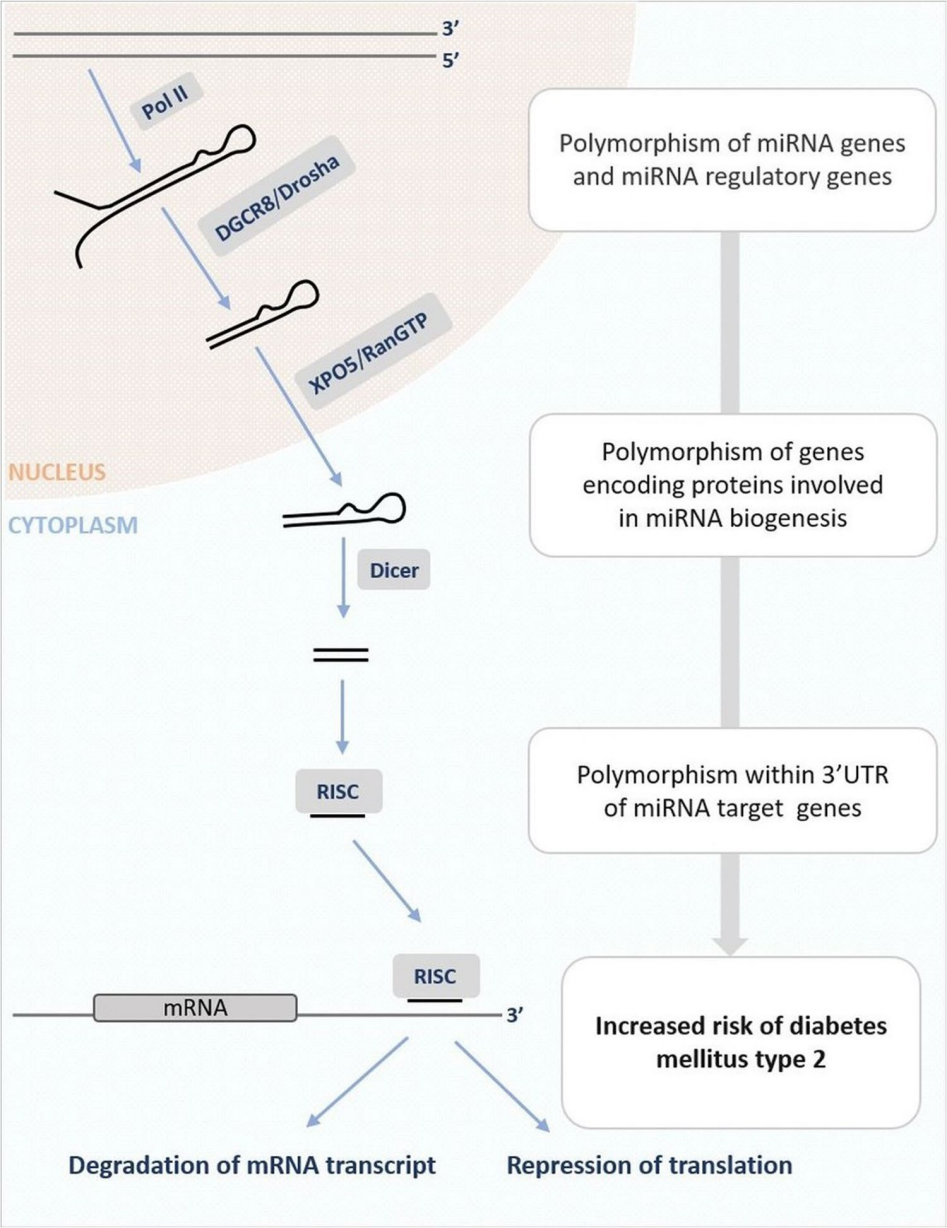
Biogenesis of miRNA via canonical pathway and polymorphism of miRNAs and their associated genes. Polymerase II (Pol II) transcribes a long primary miRNA (pri-miRNA) in the nucleus. Then, the microprocessor complex, consisting of RNA binding protein DiGeorge Syndrome Critical Region 8 (DGCR8) and Drosha, generates hairpin-like precursor miRNAs (pre-miRNAs) from pri-miRNAs. pre-miRNA is translocated into the cytoplasm by an exportin 5 (XPO5)/RanGTP complex. In the next step, Dicer process pre-miRNA into miRNA duplex. One strand of this duplex miRNA is incorporated into RISC. After that, RISC can bind into a 3’ untranslated region (3’ UTR) of target mRNA, leading to repression of translation or transcript degradation. Polymorphism of genes that are engaged in miRNA expression and maturation may lead to an increased risk of diabetes mellitus type 2 development

According to the miRBase database (v22), the human genome encodes more than 2500 miRNAs (Kozomara et al. 2019). Their genes are located in the protein-coding or non-coding part of the genome and could be transcribed by polymerase II (Lee et al. 2004) or III (Borchert et al. 2006). Maturation of miRNA requires stepwise processing (Lee et al. 2002) and takes place via a canonical (Bartel 2004) or non-canonical pathway (Miyoshi et al. 2010; Abdelfattah et al. 2014). However, the synthesis of the majority of miRNAs is via the canonical pathway (Fig. 1), in which firstly polymerase II transcribes a long primary miRNA (pri-miRNA) in the nucleus (Kim et al. 2009; O’Brien et al. 2018). This transcript has at least one hairpin structure and could be caped and polyadenylated (Cai et al. 2004). Furthermore, the microprocessor complex, consisting of RNA binding protein DiGeorge Syndrome Critical Region 8 (DGCR8) and an RNase III enzyme, Drosha, generates from pri-miRNAs the hairpin-like precursor miRNAs (pre-miRNAs), ~ 70 nt in length (Lee et al. 2003; O’Brien et al. 2018). The next step requires translocation of pre-miRNA to the cytoplasm by an exportin 5 (XPO5)/RanGTP complex (Lund et al. 2004), where the RNase III endonuclease, Dicer process pre-miRNA into miRNA duplex, ~ 22 nt in length (Han et al. 2004). One strand of this duplex miRNA usually serves as mature miRNA (Han et al. 2004) and is incorporated into an RNA-induced silencing complex, RISC (Bartel 2004). Based on miRNA sequence, RISC binds into target mRNA transcript, usually within 3’ untranslated region (3’ UTR), leading to repression of translation or transcript degradation (Kim et al. 2009). More detailed information about miRNA biogenesis, function, and mechanism of action could be found in (Bartel 2004, 2018; Krol et al. 2010; O’Brien et al. 2018).

Interestingly, miRNAs are predicted to regulate the majority of human genes (Friedman et al. 2009). Moreover, the regulatory network of miRNAs and their targets is very complex, as one miRNA could regulate multiple genes and one gene could be controlled by many miRNAs (Peter 2010). Current findings demonstrate that miRNAs exert a role in the pathogenesis of diabetes mellitus type 2 (reviewed in Yaribeygi et al. 2018; Deng and Guo 2019). For example, given that insulin resistance is the main reason for type 2 diabetes, miRNAs can modulate proteins engaged in insulin signaling, thus affecting insulin resistance and glucose homeostasis (Deng and Guo 2019). In addition, they play role in pancreatic β-cell development and survival (Filios and Shalev 2015) and even regulate lipid metabolism (Agbu and Carthew 2021). Studies conducted in various populations have shown that polymorphism in miRNA genes is associated with DM2 and diabetes complications (Elfaki et al. 2019). Moreover, Zhu et al. (2021) have reported that in sex-stratified analysis, single-nucleotide polymorphism (SNP) in miRNA genes, let-7a-1 rs13293512, and miR-27a rs895819, was associated with increased risk of DM2 in the male subjects from the Chinese population. Overall, polymorphism in miRNA genes (localized within a sequence of mature miRNA, pre-miRNA, pri-mRNA, and regulatory regions of miRNA) may lead to changes in mature miRNAs structure and their expression level resulting in dysregulated expression of miRNA’s target genes (Gong et al. 2014). The impact of genetic variants on elevated susceptibility to DM2 and its vascular complications is also observed in genes encoding proteins engaged in miRNA biogenesis which could potentially lead to changes in the miRNA processing (Wen et al. 2019). Wen et al. (2019) have found that rs13078 SNP in gene encoding DICER1 is associated with decreased susceptibility of DM2, but rs14035 SNP in gene encoding RAN resulted in a higher risk of developing macrovascular complications in the Southern Chinese Population. Given that miRNA realize their function via binding to their target mRNAs, polymorphism in 3’ UTR of mRNAs may affect miRNA binding sites leading to dysregulation of protein expression (Gong et al. 2014). For example, Zhang et al. (2013a) have proved that rs3811463 polymorphism in the let-7 target region of the Lin28 gene is associated with increased susceptibility to DM2 in females from the Chinese Han Population. In general, regardless of cause and involved mechanism, aberrant expression and function of miRNA are linked with DM2 development.

## Circulating miRNA as potential biomarkers for DM2

Apart from miRNAs that exert regulatory function inside cells, scientists have discovered extracellular, circulating miRNAs (Chim et al. 2008). It appears that miRNAs could be secreted by cells in extracellular vesicles or are transported bound with different proteins such as Argonaut proteins and high-density lipoproteins (HDL) (Nik Mohamed Kamal and Shahidan 2020). Moreover, their expression is detected in various physiological fluids and may change under different pathophysiological conditions (Weber et al. 2010). In addition, high stability, resilience to endogenous ribonucleases, along with the possibility of detection by available methods, makes circulating miRNA suitable candidates as biomarkers (Chen et al. 2008) for various diseases including cancers (George and Mittal 2010; Yu et al. 2011; Lan et al. 2015), diabetes (Guay and Regazzi 2013; Mirra et al. 2015), cardiovascular diseases (Xu et al. 2012; Kondkar and Abu-Amero 2015; Felekkis and Papaneophytou 2020), and neurodegenerative disorders (Grasso et al. 2014). A recent review and bioinformatics analysis have shown that expression of circulating miRNA miR-30a-5p, miR-30d-5p, and miR-30c-5p is associated with glucose metabolism, inflammation, platelet reactivity, and endothelial dysfunction in diabetes type 2 and underscores their utility as biomarkers for detection and progression of disease (Pordzik et al. 2019). Interestingly, among genes regulated in DM2, *PRKAR1A* (protein kinase cAMP-dependent type I regulatory subunit alpha), may be one of the most significant targets of miRNAs involved in glucose metabolism, insulin signaling, and blood coagulation. In the cells, protein kinase A is engaged in proliferation and cell growth (Pordzik et al. 2019). These findings are in line with the results of previous studies indicating that miRNA could be used as potential biomarkers for DM2 (Zhu and Leung 2015). As a result of the meta-analysis of DM2 miRNA expression profiling studies, Zhu and Leung (2015) identified eight circulating miRNAs (miR-103, miR-107, miR-132, miR-144, miR-142-3p, miR-29a, miR-34a, and miR-375) that may serve as promising biomarkers. These miRNAs regulate processes such as insulin secretion and signaling, or adipogenesis (Zhu and Leung 2015).

Considering the importance of early detection of DM2, continuous efforts are being made to find miRNAs that enable distinguishing the pre-diabetes state from the early onset of disease or identify individuals with a high risk of DM2 development (Bhatia et al. 2015) (Table 1). For example, Yan et al. (2020) built a multi-parameter diagnostic model to discriminate the impaired glucose regulation from DM2. Specifically, the expression levels of miR-148b, miR-223, miR-130a, and miR-19a in the serum may potentially be used for distinguishing patients with DM2 from that suffering from impaired glucose regulation (Yan et al. 2020). Zhang et al. (2013b) have shown that miR-126 had significantly reduced expression levels in DM2 susceptible individuals and diagnosed DM2 patients in comparison to controls which may make miR-126 useful to identify individuals with a higher risk of DM2. In turn, Jiménez-Lucena et al. (2018) in a prospective study have demonstrated that coupling the expression values of 9 miRNAs with a conventional biomarker, glycated hemoglobin (HbA1_c_), enables prediction of the DM2 development with high accuracy.

**Table 1:** Examples of circulating miRNAs that may serve as potential DM2 biomarkers

Biomarker	Sample	Diagnostic value	Population/ethnicity	Investigated Sex	References
miR-21	Plasma	Early detection of glucose imbalances	Participants from DIAPASON Study cohort, Italy	M and F	La Sala et al. (2019)
miR-126	Plasma	Prediction of susceptible individuals to DM2	Chinese Han	M and F	Zhang et al. (2013b)
miR-9, miR-28-3p, miR-29a, miR-30a-5p, miR-103, miR-126, miR-150, miR-223, miR-375	Plasma	Prediction of susceptible individuals to DM2	Patients from the CORDIOPREV Study, Spain	M, and F	Jiménez-Lucena et al. (2018)
hsa-miR-1249, hsa-miR-320b, hsa-miR-572	Plasma	Distinguish prediabetes and newly diagnosed DM2 from healthy individuals	Chinese Han	M and F	Yan et al. (2016)
miR-375	Plasma	Distinguish DM2 from healthy individuals	Chinese Kazak	M and F	Sun et al. (2014)
miR-148b, miR-223, miR-130a, miR-19a	Serum	The early diagnosis of DM2	Mongolia	M and F	(Yan et al. 2020)
miR-103b	Platelet	The early diagnosis of DM2	Chinese Han	M and F	Luo et al. (2015)
miR-503, miR-376a	Serum	Distinguish DM2 from obese DM2 patients	Spain	M and F pooled samples	Pescador et al. (2013)
miR-503, miR-138	Serum	Distinguish DM2 from obese DM2 patients	Spain	M and F pooled samples	Pescador et al. (2013)
miR-138, miR-376a, miR-15b	Serum	Distinguish obese patients from obese DM2, DM2, and healthy patients	Spain	M and F pooled samples	Pescador et al. (2013)

Abbreviations: *DM2*, diabetes mellitus type 2; *M*, male; *F*, female; *DIAPASON Study*, diabetes prediction and screening observational Study; *CORDIOPREV Study*, CORonary Diet Intervention with Olive oil and cardiovascular PREVention Study

During DM2, diabetes-induced vascular dysfunction may lead to macrovascular complications manifesting with cardiovascular diseases, as well as microvascular complications that are associated with the development of retinopathy, neuropathy, and nephropathy (Beckman and Creager 2016). Examples of circulating miRNAs that may serve as biomarkers for various diabetes vascular complications are presented in Table 2. Prevention of diabetes complications requires the selection of appropriate medication which, in an ideal situation, would be tailored to the patient. It occurs that circulating miRNAs could also be used to predict the effects of DM2 therapy (Table 3). After 1 year of treatment with glucagon-like peptide 1 receptor agonists (GLP1-RA), patients with initially higher expression levels of eight circulating miRNAs had a better response to therapy than those with lower levels (Formichi et al. 2021). In addition to the abovementioned examples, the recent results showed that circulating miRNA may be utilized to select a personalized diet model to prevent the development of DM2. In the case of patients with cardiovascular diseases, initial high miR-150 along with low miR-29a, miR-28-3p, and miR-126 in subjects on a Mediterranean diet, and low initial level of miR-145 in subjects on low-fat high-complex carbohydrate diet, was associated with a higher risk of development DM2 (Jimenez-Lucena et al. 2021). Therefore, the profile of circulating miRNAs may be also used to select a group of patients for a particular treatment in DM2 (Formichi et al. 2021) and nutritional therapy to lower the risk of disease development (Jimenez-Lucena et al. 2021).

**Table 2:** Examples of circulating miRNAs that may serve as potential biomarkers for DM2 vascular complications

Biomarker	Sample	Diagnostic value	Population/ethnicity	Investigated Sex	References
Macrovascular complications					
miR-181c-5p	HDL fraction of plasma	Distinguish DM2 with peripheral artery disease from DM2 and healthy patients	Indigenous Australian	M	Morrison et al. (2021)
miR-130	Serum	Distinguish DM2 with CAD from CAD patients	China	M and F	Yuan et al. (2019)
miR-126	Peripheral whole blood	Distinguish DM2 and DM2 with CAD from healthy patients	Arabian	M and F	Al-Kafaji et al. (2017)
miR-126	Peripheral whole blood	Distinguish DM2 with CAD from DM2	Arabian	M and F	Al-Kafaji et al. (2017)
miR-1, miR-133	Whole blood	Early detection of CAD in DM2 patients	Arabian	M and F	Al-Muhtaresh et al. (2019)
miR-126, miR-210	Plasma	Distinguish DM2 with CAD from DM2	Egypt	M and F	Amr et al. (2018)
miR-1, miR-21	Serum	Prediction of acute heart failure in DM2 patients	Turkey	M and F	Al-Hayali et al. (2019)
Microvascular complications					
miR-1281	Serum	Early detection of diabetic retinopathy in DM2 patients	Caucasian	M and F	Greco et al. (2020)
miR-25-3p, miR-320b, miR-495-3p	Plasma exosomes	Diagnosis of diabetic retinopathy in DM2 patients	Italy	M and F	Santovito et al. (2021)
miR-21	Plasma	Indicating the severity of diabetic retinopathy in DM2 patients	China	M and F	Jiang et al. (2017)
miR-21-5p, miR-30b-5p	Urinary exosomes	Indicating renal function	European (Ireland)	M, and F	(Zang et al. 2019)
miR-196a	Urine	Prognostic biomarker of renal fibrosis in patients with diabetic nephropathy	China	M, and F	An et al. (2020)
miR-128a, miR-155, miR-499a	PBMCs	Prediction of neuropathic complications in DM2	Italy	M, and F	Ciccacci et al. (2020)

Abbreviations: *DM2*, diabetes mellitus type 2; *M*, male; *F*, female; *CAD*, coronary artery disease; *DR*, diabetic retinopathy; *PBMCs*, peripheral blood mononuclear cells

**Table 3:** Examples of circulating miRNAs that may serve for the selection of patients that will respond to particular DM2 treatment and therapy

Biomarker	Sample	Diagnostic value	Population/ethnicity	Investigated Sex	References
miR-24-3p, miR-126-3p, miR-21-5p, miR-15a-5p, miR-223-3p, miR-378-3p, miR-375-3p, miR-146-5p	Plasma	Prediction of response to GLP1-RA treatment in DM2 patients	Italy	M and F	Formichi et al. (2021)
miR-378, miR-126-3p, miR-223	Plasma	Disease staging and predicting response to sitagliptin treatment in DM2 elderly patients	Italy	M and F pooled	Catanzaro et al. (2018)
miR-29a, miR-28-3p, miR-126, miR-150, miR-145	Plasma	Selection of diet therapy to prevent DM2 development in coronary heart disease patients	Patients with cardiovascular disease from the CORDIOPREV Study, Spain	Not applicable	Jimenez-Lucena et al. (2021)

Abbreviations: *DM2*, diabetes mellitus type 2; *M*, male; *F*, female; *GLP1-RA*, glucagon-like peptide 1 receptor agonists; *CORDIOPREV Study*, CORonary Diet Intervention with Olive oil and cardiovascular PREVention Study

## Sex difference in circulating miRNA in DM2

Scientists have discovered the sex-biased expression of microRNA in many pathological conditions, including metabolic diseases (Sharma and Eghbali 2014). For example, Wang et al. (2013) demonstrated that elevated expression level of circulating let-7 g and miR-221 was associated with metabolic syndrome. This disorder is characterized by clinical features such as abdominal adiposity, insulin resistance, hypertension, dyslipidemia, inflammation, and a prothrombotic state, and is linked with an increased risk of DM2 development. Further analysis revealed a sex-specific expression pattern of both investigated miRNAs. Their level was elevated in the serum of women with metabolic syndrome in comparison to healthy females, while this pattern was not reported in men (Wang et al. 2013).

Surprisingly, not every study takes into account sex differences in the circulating miRNA expression of DM2 patients, even though sex hormones, as well as X-linked genes, may potentially regulate their expression (Corral-Fernández et al. 2013; Sharma and Eghbali 2014). Interestingly, results obtained by Prabu et al. (2015) showed that there are associations between the sex of patients with pre-diabetic or DM2 state and circular miRNA expression. Moreover, the aberrant expression of some circulating miRNA may be found only in one sex. In the beginning, they noticed that in Asian Indian female and male patients analyzed together, miR-142-3p were not differentially expressed between pre-diabetes or DM2, and controls. In turn, when the analysis of miRNA expression was sex stratified, an increased level of the abovementioned miRNA was shown in the samples of pre-diabetic women versus healthy samples. In addition, circulating miR-423-5p and miR-128 altered expression was confirmed in comparison of pre-diabetes with control only in men or women, respectively. Comparing DM2 with the control samples has shown that an increased level of miR-374a-5p was found only in females (Prabu et al. 2015). More recent results of the research also emphasized the sex differences in circulating miRNA expression level but in the population of Israeli Arab and Jewish patients. Although the studied group was genetically heterogenous, Meerson et al. (2019) have found that expression levels of miR-146a-5p, miR-16–2-3p, miR-126-5p, miR-30d, and miR-423 may serve as biomarkers to distinguish early from complicated DM2. Further sex-stratified analysis showed their better diagnostic accuracy for men than women (Meerson et al. 2019). Last but not least research demonstrates that the sex-specific expression level of miRNA could be analyzed together with other markers to indicate diabetes complications. Heart failure with preserved ejection fraction (HFpEF) is an example of diabetes microvascular complications that have more prevalence in women and are frequently undiagnosed at the early stage of development (Florijn et al. 2020). Interestingly, the elevated expression level of a protein marker of microvascular injury angiopoietin-2 (Ang-2) only in diabetic females with HFpEF along with lower expression levels of miR-224 and miR-452 in diabetic males with HFpEF (Florijn et al. 2020) shows their potential to serve as sex-biased biomarkers. In summary, all the mentioned results demonstrate the need for the development of sex-specific diagnostic strategies in diabetes mellitus type 2, as well as show the possibility of using circulating miRNAs as sex-biased biomarkers. In addition, recent studies have shown the role of microRNAs, identified as candidates for sex-dependent biomarkers, in different types of cells involved in DM2 pathogenesis (Table 4). For example, they are engaged in cellular processes such as apoptosis, proliferation, oxidative stress, and regulation of lactate transport (Chen et al. 2021a; Blum et al. 2019; Luo et al. 2020; Xiao et al. 2019; Zhao et al. 2021).

**Table 4:** Role of circulating microRNA with sex-biased expression in different types of cells involved in DM2

microRNA	Type of cells	Signaling pathway	Biological function	References
miR-142-3p	HK-2	miR-142-3p/BOD1	Inhibition of HG-induced apoptosis and oxidative stress	Zhao et al. (2021)
miR-423-5p	ARPE-19	NFE2/miR-423-5p/TFF1	Regulation of HG-induced apoptosis	Xiao et al. (2019)
miR-423-5p	HK cells	XIST/miR-423-5p/HMGA2	Regulation of apoptosis and proliferation in HG-treated HK cells	Chen et al. (2021a)
miR-146a-5p	INS-1 cells	LncRNA PTGS2/miR-146a-5p/RBP4	miR-146a-5p may be involved in the inhibition of INS-1 cells dysfunction generated by RBP4	Chen et al. (2021b)
miR-423	HUVECs	There may be a link between miR-423, VEGF, and eNOS expression	miR-423 may be involved in the regulation of vascular proliferation in diabetes retinopathy	Blum et al. (2019)
miR-1281	ARPE-19	miR-1281 could potentially regulate VEGF expression	miR-1281 may exert a pathogenic role in the development of diabetic retinopathy	Greco et al. (2020)
miR-425-5p	HUVECs	NF-κB/miR-425-5p/MCT4	Regulation of lactate transport in HG and IL-1β-treated endothelial cells	Luo et al. (2020)

Abbreviations: *HK-2 cells*, human renal tubular epithelial cells; *BOD1*, biorientation of chromosomes in cell division 1; *HG*, high glucose; *ARPE-19*, adult retinal pigment epithelial cell line-19; *NFE2*, nuclear factor-erythroid 2; *TFF1*, trefoil factor 1; *HK cells*, human kidney cells; *XIST*, X-inactive-specific transcript; *HMGA2*, high mobility group protein A2; *RVEC cells*, human retinal vascular endothelial cells; *INS-1 cells*, mouse pancreatic β cell cells; *RBP4*, retinol-binding protein 4; *HUVEC*, human umbilical vein endothelial cells; *ARPE-19*, human retinal pigment epithelial cells; *MCT4*, monocarboxylate transporter 4

However, there is also an interesting biomarkers searching strategy that focuses on those miRNAs which are expressed in both sexes. Considering that the previous studies suggested greater prevalence and severity of diabetic retinopathy (DR) in males with early diagnosed DM2 (Ozawa et al. 2015), Greco et al. (2020) included only male subjects in the initial screening of potential circulating miRNA biomarkers. Subsequently, they validated the aberrant expression of selected miRNAs in the samples obtained from both male and female subjects that led to findings that in both sexes the most up-regulated miRNA is miR-1281 in comparison to controls. Further analysis confirmed that circulating miR-1281 may serve as a potential biomarker for early diagnosis of DR in males and females (Greco et al. 2020).

## A potential mechanism underlying sex difference in circular miRNA expression

To date, mechanisms underlying different expressions of miRNA between females and males are not fully recognized. Based on the literature review, Florijn et al. proposed two mechanisms that may be involved in the sex-biased expression of circulating miRNA: (1) incomplete X-chromosome inactivation leading to biallelic expression of miRNA, and in addition, (2) regulation of miRNA transcription and processing by estrogens (Florijn et al. 2018; 2020). However, these processes may only partially explain the differential expressions of miRNA between sexes. The epigenetic mechanisms also contribute to the regulation of miRNA expression (Bianchi et al. 2017). For example, Sun et al. (2014) suggested that hypomethylation of miR-375 gene promoter may be associated with its overexpression in the plasma of DM2 patients. Another study has shown that in pancreatic islets, methylation of 160 sites annotated to miRNA on chromosome X and only 3 autosomal miRNA genes, was sex dependent. Further analysis indicated that miR-660 and miR-532, encoded on chromosome X, displayed lower methylation levels and higher expression levels in females pancreatic islets (Hall et al. 2014); therefore, the different methylation patterns of miRNA genes in females and males may contribute to the observed sex-dependent level of circulating miRNAs. Interestingly, Ciccacci et al. (2020) suggested that rs11888095 SNP in gene encoding miR-128a may contribute to its higher expression in peripheral blood mononuclear cells from patients with diabetic polyneuropathy. In addition, the recent results from miRNA gene and miRNA target gene polymorphic variants analyses showed that even a single-nucleotide polymorphism could have different impacts between sexes (Zhang et al. 2013a; Zhu et al. 2021). Notably, the effects of polymorphic variants of miRNA genes may be modulated by interactions with polymorphic variants of other miRNAs or by the influence of environmental factors such as smoking or dyslipidemia (Zhu et al. 2021). Considering, the abovementioned results, additional studies are needed to elucidate the potential role of genetic variants and epigenetic mechanisms in sex-dependent aberrant expression of circulating miRNA in DM2.

## Conclusion and future perspective

Circulating miRNAs are promising candidates as biomarkers for screening and diagnosis of early onset, progression, and complications of DM2 or even for the selection of individually tailored medications and therapy. However, some studies have reported discrepancies in the analyzed expression level of circulating miRNA and their utility as biomarkers in different populations (Bhatia et al. 2015; Yu et al. 2020). The establishment and validation of protocols for detection and assessment of the clinical value of investigated circulating miRNA will enable overcoming these problems. Moreover, recent findings indicate that sex may affect the results obtained in biomarkers research for DM2. The approach in which males and females are analyzed as one group may lead to overlooking some important biomarkers or finding biomarkers more precisely predicting diseases in one sex. A better understanding of the mechanism underlying sex difference in DM2 susceptibility, development, and progression would enable the selection of the most accurate circulating miRNA biomarkers and transfer those findings into clinical practice.

## Data Availability

N/A.

## Code availability

N/A.
